# Inhibition of mitotic kinase Mps1 promotes cell death in neuroblastoma

**DOI:** 10.1038/s41598-020-68829-y

**Published:** 2020-07-20

**Authors:** Sonia Simon Serrano, Wondossen Sime, Yasmin Abassi, Renée Daams, Ramin Massoumi, Mohamed Jemaà

**Affiliations:** Department of Laboratory Medicine, Translational Cancer Research, Faculty of Medicine, Lund University, 22381 Lund, Sweden

**Keywords:** Paediatric cancer, Apoptosis

## Abstract

Neuroblastoma is the most common paediatric cancer type. Patients diagnosed with high-risk neuroblastoma have poor prognosis and occasionally tumours relapse. As a result, novel treatment strategies are needed for relapse and refractory neuroblastoma patients. Here, we found that high expression of Mps1 kinase (mitotic kinase Monopolar Spindle 1) was associated with relapse-free neuroblastoma patient outcomes and poor overall survival. Silencing and inhibition of Mps1 in neuroblastoma or PDX-derived cells promoted cell apoptosis via the caspase-dependent mitochondrial apoptotic pathway. The mechanism of cell death upon Mps1 inhibition was dependent on the polyploidization/aneuploidization of the cells before undergoing mitotic catastrophe. Furthermore, tumour growth retardation was confirmed in a xenograft mouse model after Mps1-inhibitor treatment. Altogether, these results suggest that Mps1 expression and inhibition can be considered as a novel prognostic marker as well as a therapeutic strategy for the treatment of high-risk neuroblastoma patients.

## Introduction

Neuroblastoma is the most common extra-cranial solid tumour of childhood^1,2^ causing approximately 10% of all childhood cancer-related deaths^3,4^. Neuroblastoma is a very heterogeneous tumour compared to other cancers. It can regress spontaneously, however, in certain cases, can undergo treatment resistance^5,6^. The International Neuroblastoma Risk Group (INRG) classifies neuroblastoma tumours into very low-risk, low-risk, intermediate-risk, and high-risk^7,8^. Identification of the high-risk group is important for the development of personalised medicine for neuroblastoma patients with poor prognosis. Good prognosis has been observed in children with low- or intermediate-risk after chemotherapy in combination with surgical intervention. High-risk neuroblastoma represent 40% of all diagnosed cases, and these patients have poor prognosis harbouring tumour relapse and chemoresistance, and only 50% will attain long-term survival^9,10^. *MYCN* gene amplification has been observed in less than half of the high-risk tumours^11^. In non-MYCN high-risk neuroblastoma, point mutations in TP53 and Anaplastic Lymphoma Kinase (ALK) gene mutation have been observed^12^ in less than 10% of neuroblastomas^13^.

Recently, targeting cell cycle and in particular mitosis has been proposed as an alternative therapeutic strategy for cancer treatment^14^. Spindle Assembly Checkpoint or SAC generally monitor proper mitosis by controlling the correct attachment of the chromosomes to the microtubule spindle apparatus via their kinetochores^15^. Once the chromosomes are fully arranged on the metaphase plate, the SAC is turned off, and chromosome segregation as well as cell division can be engaged^16^.

The Mps1 kinase (Monopolar spindle1) is an important regulator of the SAC and it phosphorylates target proteins principally on tyrosines, serines, and threonines^17^. The most important function of Mps1 is to ensure proper biorientation of sister chromatids on the mitotic spindle at kinetochores. In early mitosis, Mps1 resolves the kinetochores–microtubules miss-attachments^15,18^. Mps1 is overexpressed in several tumours, including malignant fibrous histiocytoma^19^, pancreatic cancer^20^, glioblastoma^21^, breast cancer^22^, and thyroid cancer^23^. In breast cancer, the expression of Mps1 has been shown to be correlated with a high histologic grade, tumour aggressiveness, aneuploidy and chromosomal instability (CIN)^24,25^. However, the role of Mps1 in neuroblastoma is unknown.

A growing number of Mps1 inhibitors have been developed recently. Large board kinase inhibitor, Reversine (2-(4-morpholinoanilino)-6-cyclohexylaminopurine), is the most common Mps1 inhibitor used in cell biology research. Reversine was described originally as Aurora B inhibitor and in 2010 introduced as a specific inhibitor of Mps1 by Stefano Santaguida and co-workers^26^. Structural comparison of Reversine bound to Mps1 and Aurora B confirmed direct binding affinity to Mps1 with a two-fold higher affinity to Mps1 than Aurora B^27^. Mps-BAY2a (an imidazopyrazine) *N*-cyclopropyl-4-{8-[(2-methylpropyl)amino]-6-(quinolin-5-yl)imidazo[1,2-a]pyrazin-3-yl}benzamide is a synthetic compound developed for clinical studies. Mps-BAY2a showed a restricted inhibitory effect on a panel of 220 human kinases compared to Reversine^25^.

Neuroblastoma relies largely on DNA damaging agents and spindle poisons for chemotherapy. The identification of new targets can improve survival of high-risk neuroblastoma patients. Here, we report that Mps1 overexpression is a poor prognostic marker for neuroblastoma patients and that Mps1 inhibition provokes cancer cell death via polyploidization and mitotic catastrophe.

## Results

### Mps1 expression as a novel prognostic marker for human neuroblastoma

**Figure 1:**
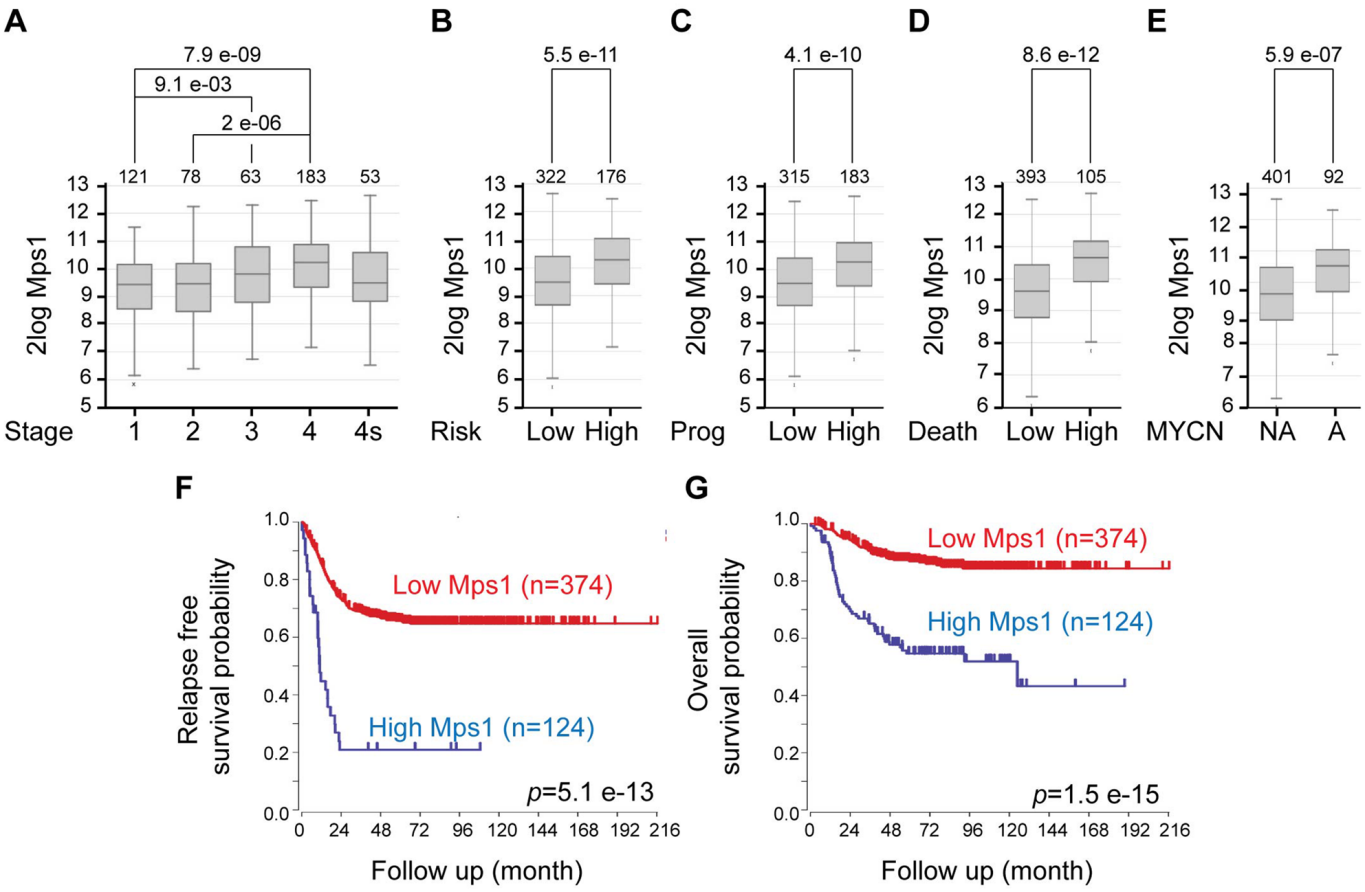
Mps1 expression correlates with aggressive tumors and predicts clinical outcome in neuroblastoma patients. (**A**–**E**) Box plots of Mps1 expression relative to tumor stage (**A**), risk classification (**B**), tumor progression (**C**), death from the tumor (**D**) and MYCN status (**E**). (**F**,**G**) Kaplan–Meier curves reporting patient’s event-free survival (**F**) and overall survival (**G**) probability with respect to Mp1 expression.

To investigate whether Mps1 expression is altered in neuroblastoma, we took advantage of the R2-Genomics analysis and visualisation platform (https://r2.amc.nl) and explored multiple neuroblastoma gene expression data sets for Mps1 expression (GSE49710 in Fig. 1 and GSE45547, GSE16476 and E-TABM-38 in Figure S1). We found that Mps1 expression is highly correlated with advanced stages using the International Neuroblastoma Staging System (INSS) (Fig. 1A and Fig. S1A–C). Moreover, Mps1 overexpression was associated with tumour progression, tumour risk and patient mortality (Fig. 1B–D). MYCN is often amplified in aggressive neuroblastomas and highly correlated with advanced disease^28^. Analysis of neuroblastoma patients showed that Mps1 expression was significantly higher in MYCN amplified neuroblastoma compared to non-amplified (Fig. 1E and Fig. S1A–C). Moreover, Kaplan–Meier curves revealed that high Mps1 expression was associated with poor overall and relapse-free survival (Fig. 1F,G and Fig. S1A–C). These data strongly support Mps1 expression as a novel prognostic marker for human neuroblastoma.

### Mps1 inhibition provokes the mitochondrial pathway of apoptosis in neuroblastoma cells

**Figure 2:**
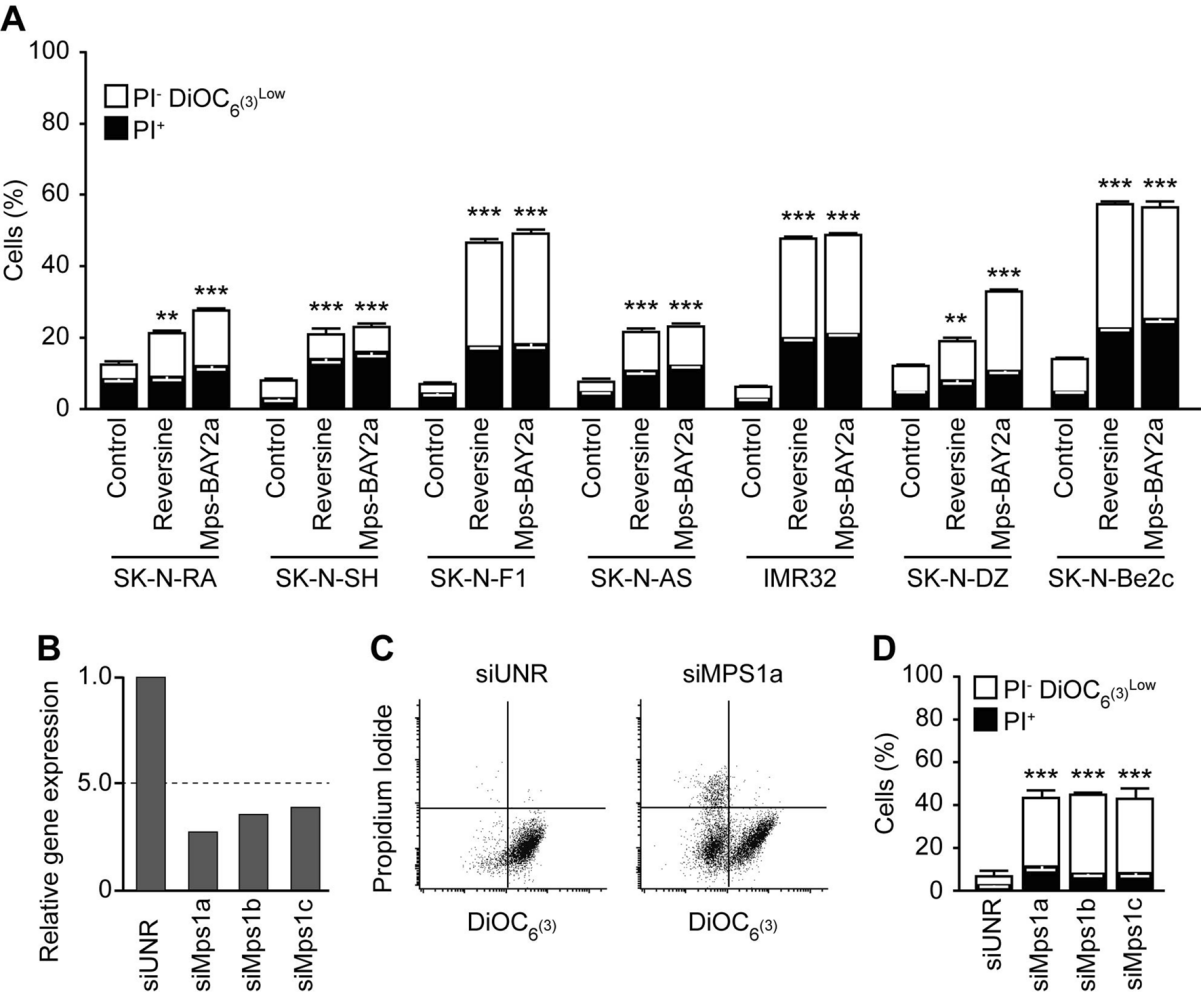
Mps1 inhibition kills neuroblastoma cells. (**A**) Neuroblastoma cells were treated for 72 h with DMSO as control, 0.3 μM reversine or 1 μM Mps-BAY2a and then co-stained with the vital dye propidium iodure (PI) and the mitochondrial membrane potential (Δψm)-sensing dye DiOC6(3) for the evaluation of cell death-associated parameters by cytofluorometry. White and black columns depict the percentage of dying (PI^−^DiOC6(3)^low^) and dead (PI^+^) cells, respectively. (**B**–**D**) SK-N-Be2c neuroblastoma cells were transfected with an unrelated siRNA (siUNR) or three specific siRNAs directed against Mps1 (siMps1a, siMps1b and siMps1c). Upon 72 h, cells were collected and lysed for real time PCR analysis (**B**). Alternatively, cells were subjected to the determination of the cell death–associated parameters by flow cytometry upon co-staining with the propidium iodide (PI) and DiOC_6_(3) dyes. Representative plots are showed in panel (**C**) while quantitative data are displayed in panel (**D**). White and black columns illustrate the percentage of dying (PI^−^ DiOC_6_(3)^low^) and dead (PI^+^) cells, respectively. Data are reported in SEM n = 3. **(p < 0.01) and ***(p < 0.001) indicates significant difference from the DMSO control treatment or siUNR transfected cells (ANOVA).

**Figure 3:**
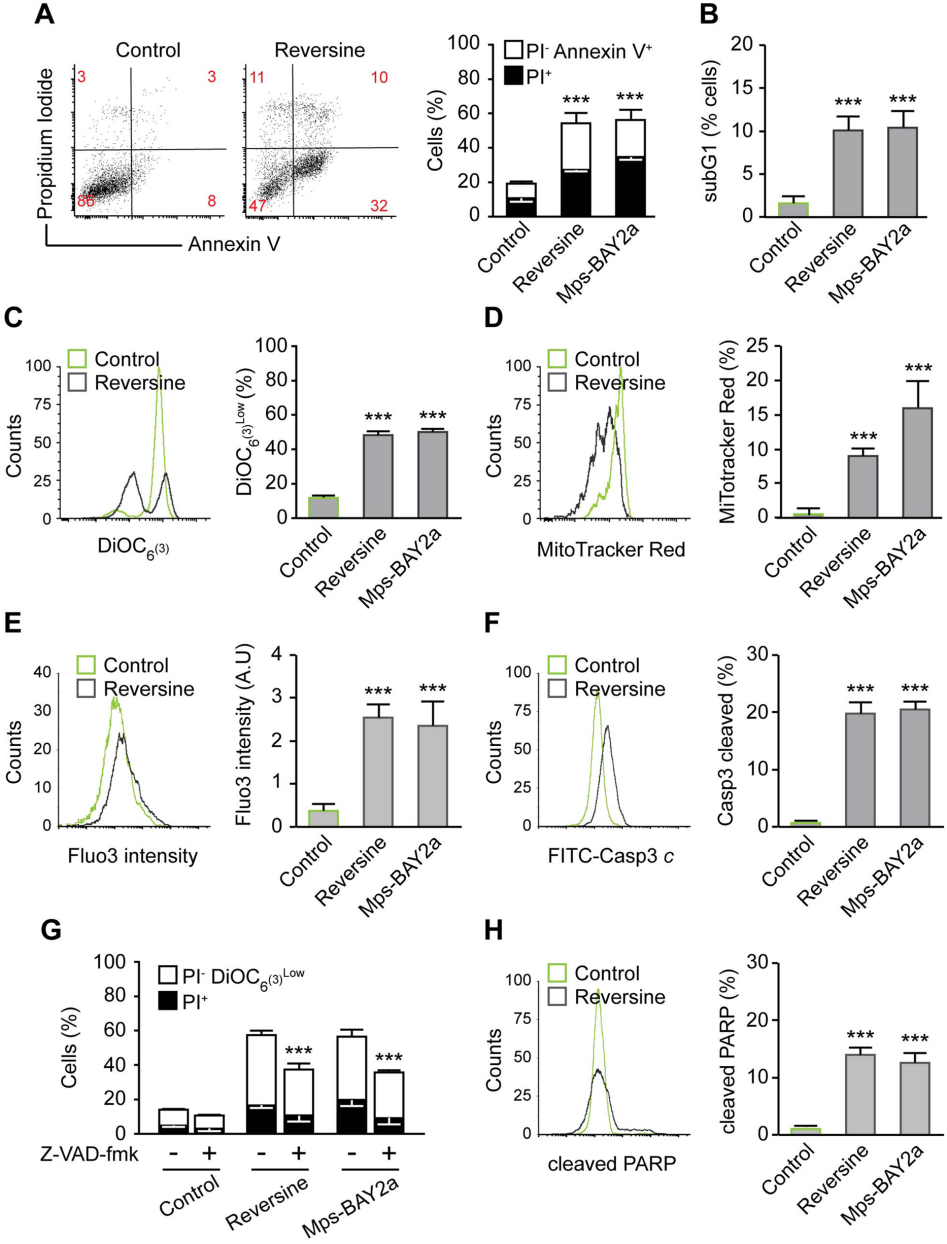
Mps1 inhibition kills neuroblastoma cells via the mitochondrial pathway of apoptosis. The toxicity of Mps1 inhibitors Reversine and Mps-BAY2a at 72 h was evaluated by flow cytometry upon staining with the cell death-associated parameters dyes and antibodies. (**A**) SK-N-Be2c neuroblastoma cells were treated for 72 h with DMSO as control, 0.3 μM Reversine or 1 μM Mps-BAY2a and then co-stained with the vital dye propidium iodide (PI) and the FITC-conjugated Annexin V for the detection of phosphatidylserine exposure. Representative dot plot (Reversine treatment) and quantitative data are reported. White columns depict the percentage of dying cells (PI^−^Annexin V^+^) while black columns illustrate dead cells (PI^+^). Numbers indicate the percentage of cells found in each gate. (**B**) DMSO, Reversine and Mps-BAY2a treated cells were fixed with cold 75% ethanol and labeled with propidium iodide (PI) as DNA dye, for the quantification of the subG1 apoptotic population of the cell cycle. Quantitative data are reported. (**C**) Cells were stained with the mitochondrial membrane potential (Δψm)-sensing dye DiOC6(3). Representative histogram of Reversine vs. DMSO treated cells is shown and quantitative data of the signal shift are represented. Control cells treated with DMSO are depicted in green while Mps1 inhibitor treated ones are depicted in grey. (**D**) Cells were stained with the MitoTracker Red dye for the quantification of mitochondrial accumulation dependent upon membrane potential. Representative histogram of Reversine vs. DMSO treated cells is shown and quantitative data of the dye intensity are reported. Control cells treated with DMSO are depicted in green while Mps1 inhibitors treated ones are depicted in grey. (**E**) Cells were stained with the calcium dye Fluo-3 for quantification of the cytosolic Ca^2+^ concentration by flow cytometry. Representative histogram of Reversine vs. DMSO treated cells is shown and quantitative data of the dye intensity are reported. Control cells treated with DMSO are depicted in green while Mps1 inhibitors treated ones are depicted in grey. (**F**) DMSO, Reversine and Mps-BAY2a treated cells were fixed with cold 75% ethanol and labeled with the FITC-conjugated Casp3 for the cleaved caspase-3 detection. Representative histogram of Reversine vs. DMSO treated cells is shown and quantitative data are reported. Control cells treated with DMSO are depicted in green while Mps1 inhibitors treated ones are depicted in grey. (**G**) Effects of Pan Caspase inhibitor Z-VAD-fmk on Mps1 inhibitors induced cell death. SK-N-Be2c neuroblastoma cells were treated for 72 h with Mps1 inhibitors alone or in combination with 25 μM Z-VAD-fmk followed by DiOC6(3)/PI co-staining. Quantitative data are represented. White and black columns depict the percentage of dying (PI^−^DiOC6(3)^low^) and dead (PI^+^) cells, respectively. (**H**) DMSO, Reversine and Mps-BAY2a treated cells were fixed with cold 75% ethanol and labeled with the cleaved PARP antibody. Representative histogram of Reversine vs. DMSO treated cells is shown and quantitative data are reported. Control cells treated with DMSO are depicted in green while Mps1 inhibitors treated ones are depicted in grey. Data are reported in SEM n = 3. ***(p < 0.001) indicates significant difference from the DMSO control treatment (ANOVA).

To evaluate the effect of Mps1 inhibition on neuroblastoma cells, we used different cellular phenotypic variants of neuroblastoma cell lines, including neuroblastic (N-type: SK-N-F1, SK-N-RA, and IMR-32) or more malignant neuroblastoma cells, the intermediate type (I-type: SK-N-Be2c). These cells differ in MYCN amplification and ALK and TP53 status (Table 1). Moreover, the majority of these cells are isolated from metastatic tumours and they are insensitive to several common agents used in classic chemotherapy, including cyclophosphamide, doxorubicin and vincristine. We first treated the selected neuroblastoma cell lines with DMSO as a control and two different Mps1 inhibitors (Reversine and Mps-BAY2a) for 72 h. First the effect of Reversine (0.3 μM) and Mps-BAY2a (1 μM) on cell death was evaluated by flow cytometry using co-staining with the mitochondrial inner transmembrane potential (Δψm) sensitive dye DiOC_6_(3) and the vital dye propidium iodide (PI) that leads to the identification of dying (DiOC_6_(3)^low^ PI^−^) as well as dead (PI^+^) cells (Fig. 2A). All cell lines used were significantly sensitive to Mps1 inhibition. We decided to focus our investigation on SK-N-Be2c neuroblastoma cells, since this cell line accumulates MYCN amplification, TP53 mutation, and chemoresistance, but also has a high sensitivity to Mps1 inhibition. First, to avoid any off-target effects of Reversine or Mps-BAY2a, we used three different small interfering (si)RNAs specifically directed against the kinase (siMps1) (Fig. 2B) and confirmed the cytotoxic effect of depleting Mps1 in SK-N-Be2c neuroblastoma cells compared to the siRNA control (Fig. 2C,D). To study the mechanism of cell toxicity induced by Mps1 inhibition, we performed several cell death assays using flow cytometry. The cells were treated with DMSO as a control and Reversine or Mps-BAY2a as Mps1 inhibitors for 72 h. We first analysed the cell size based on their forward scatter properties using flow cytometry. We found that Mps1 inhibition provokes cell shrinking, which is the hallmark of cells undergoing apoptosis (Fig. S2A). Cells were also co-stained with the vital dye PI and the apoptotic marker Annexin-V for scrambling and phosphatidylserine exposure to the cell surface. We found that treated cells underwent apoptosis compared to the control (Fig. 3A). Moreover, analysis of the cell cycle profile showed the appearance of a sub-diploid population (Sub G1) corresponding to apoptotic bodies and fragmented DNA in the treated cells compared to the control cells (Fig. 3B). To understand via which pathway cells undergo apoptosis, we performed mitochondrial assays. We first measured the mitochondrial transmembrane potential (Δψm) of the control and the Mps1 inhibitors-treated cells using the sensor DiOC_6_(3) (Fig. 3C) and the MitoTracker Red (MTR) dye (Fig. 3D). We found that Mps1 inhibitors-treated cells significantly lost their mitochondrial potential compared to the control condition. The drop of mitochondrial membrane potential as a consequence of its hyperpolarisation is a hallmark of intrinsic apoptosis^29^. To further confirm that the mitochondrial pathway of apoptosis is engaged after Mps1 inhibition, we measured the intracellular calcium (Ca^2+^) concentration. Indeed, Ca^2+^ is involved in regulating mitochondrial morphology and the release of pro-apoptotic proteins^30^. The increase of cytosolic Ca^2+^ concentration is followed by an increase of mitochondrial Ca^2+^ concentration, which induces the mitochondrial permeability transition pore (MPTP) with the consequent release of cytochrome *c* and apoptotic response^31^. We used the Fluo-3/AM dye to track cytosolic Ca^2+^ activity and found that treated cells had a higher calcium concentration compared to the DMSO control (Fig. 3E). Intrinsic apoptosis is subdivided into caspase-dependent and caspase-independent sub-pathways. Using a specific antibody, we found that Mps1 inhibition induces caspase-3 cleavage and activation in neuroblastoma cells (Fig. 3F). Moreover, the co-administration of the broad-spectrum caspase inhibitor Z-Val-Ala-Asp fluoromethylketone (Z-VAD-fmk) significantly reduced the death of SK-N-Be2c neuroblastoma cells responding to the inhibition of Mps1 (Fig. 3G). Further confirming the role of caspases in the execution of apoptosis, neuroblastoma cells treated with Reversine or Mps-BAY2a manifested the apoptosis-associated cleavage of poly (ADP-ribose) polymerase (PARP) (Fig. 3H). Altogether, these results suggest that Mps1 inhibition in neuroblastoma cells induces cell death via the activation of the mitochondrial and caspase-dependent pathway of apoptosis. To further study the role of Mps1 inhibition in other cell death subroutines, we decided to investigate necroptosis and autophagy in treated cells. Indeed, necroptosis was, until recently, considered as apoptosis or programmed cell death^32^. As a cell death subroutine, necroptosis shares with necrosis several hallmarks, such as early loss of mitochondrial membrane integrity and the rupture of the plasma membrane after cellular swelling^33^. Necrostatin-1 is a specific inhibitor of the necrosome leading to an inhibition of necroptosis^34^. The co-administration of 50 or 100 μM Necrostatin-1 with Mps1 inhibitors failed to reduce cell death and thus excluding any role of Mps1 inhibition to induce regulated necrosis (Fig. S2B). Autophagosome formation was shown to be upregulated upon Mps1 depletion^35^, thus we investigated autophagy in SK-N-Be2c neuroblastoma cells treated with Reversine or Mps-BAY2a. We used Acridine Orange staining and flow cytometry. Acridine Orange is a cell-permeable green dye that shifts in to red fluorescence when it get locked-in acidic vesicular organelles like autophagosomes. This makes Acridine Orange staining a reliable method to assess the volume of acidic vesicular organelles, which increases upon autophagy induction^36^. We assessed the red-to-green fluorescence intensity ratio (Red/Green) to quantify the Acridine-Orange stained cells with flow cytometry^36^. We found that Mps1 inhibition with Reversine or Mps-BAY2a did not induce any autophagy in neuroblastoma cells (Fig. S2C).

**Table 1:** Different cell lines used in the study.

Cells	MYCN	TP53 mutation	ALK mutation	Reference
SK-N-Be2c	MYCN amplification	C135F	WT	^61^
SK-N-DZ	MYCN amplification	R110L	WT	^61^
SK-N-RA*	Normal	WT	F1174L	^62^
SK-N-F1	Normal	M246R	WT	^61^
IMR32	MYCN amplification	WT	WT	^61^
SK-N-SH	Normal	WT	F1174L	^61^
SK-N-AS	Normal	H168R	WT	^61^
*RA subclone of SH				^63^

### Mps1 abrogation induces the polyploidization of neuroblastoma cells and accumulation of mitotic defaults

**Figure 4:**
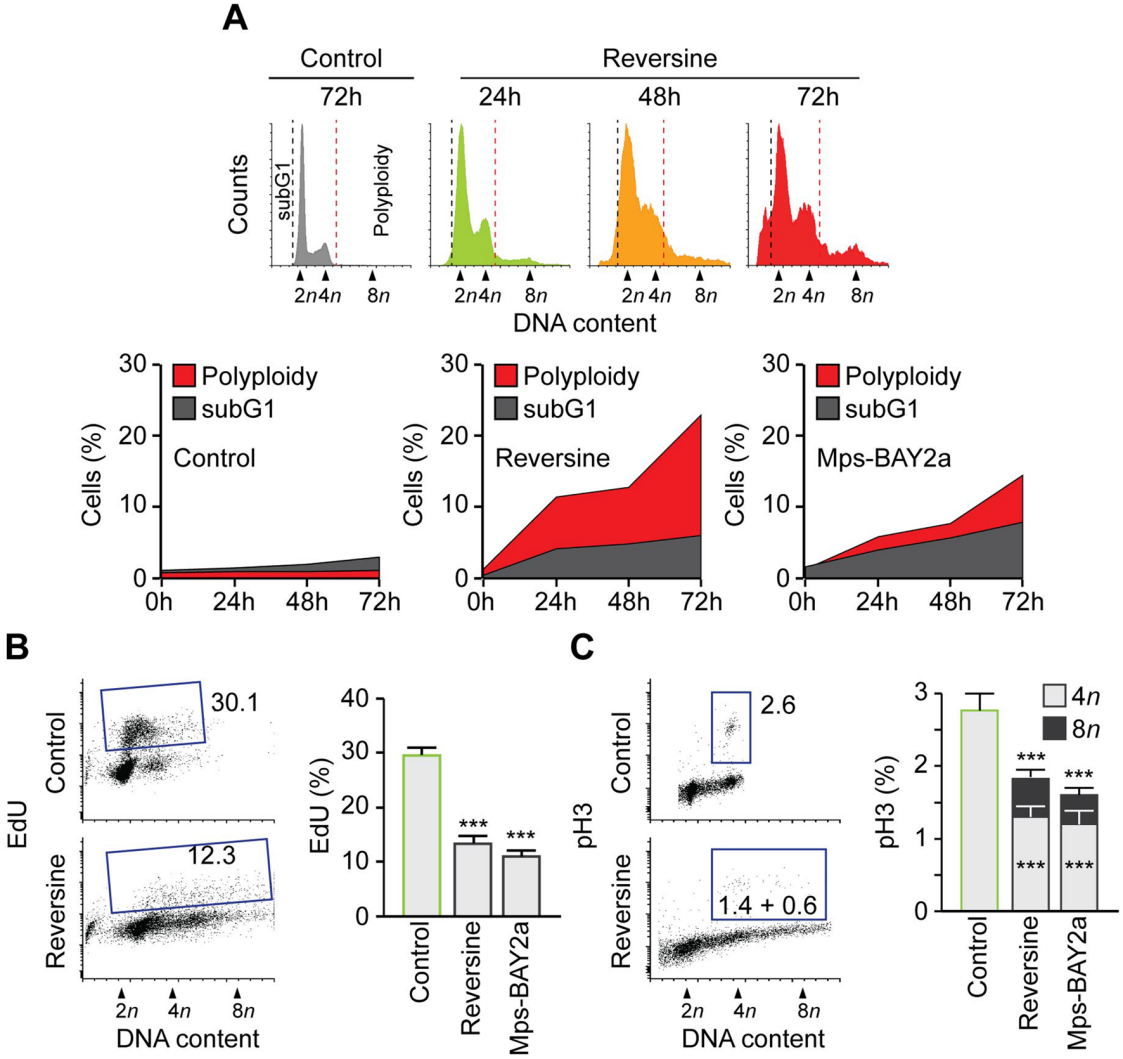
Mps1 inhibition induces polyploidy in neuroblastoma cells. (**A**) SK-N-Be2c neuroblastoma cells were treated with DMSO or Mps1 inhibitors for 24 h, 48 h and 72 h. Cells were fixed and labeled for cell cycle analysis. Representative histograms (control and Reversine) and quantitative data of polyploid and subG1 fraction are reported. (**B**) SK-N-Be2c neuroblastoma cells were treated with DMSO or Mps1 inhibitors for 72 h. Cells were labeled for 1 h with EdU before being fixed. EdU incorporation was calculated by flow cytometry. Representative plots (control and Reversine) and quantitative data for both Reversine and Mps-BAY2a are reported. (**C**) Fixed cells were stained for an antibody specific for phosphorylated histone 3 (pH3), which is a mitosis-specific marker. Representative plots and quantitative data for both Reversine and Mps-BAY2a are reported. Data are reported in SEM n = 3. ***(p < 0.001) indicates significant difference from the DMSO control treatment (ANOVA).

**Figure 5:**
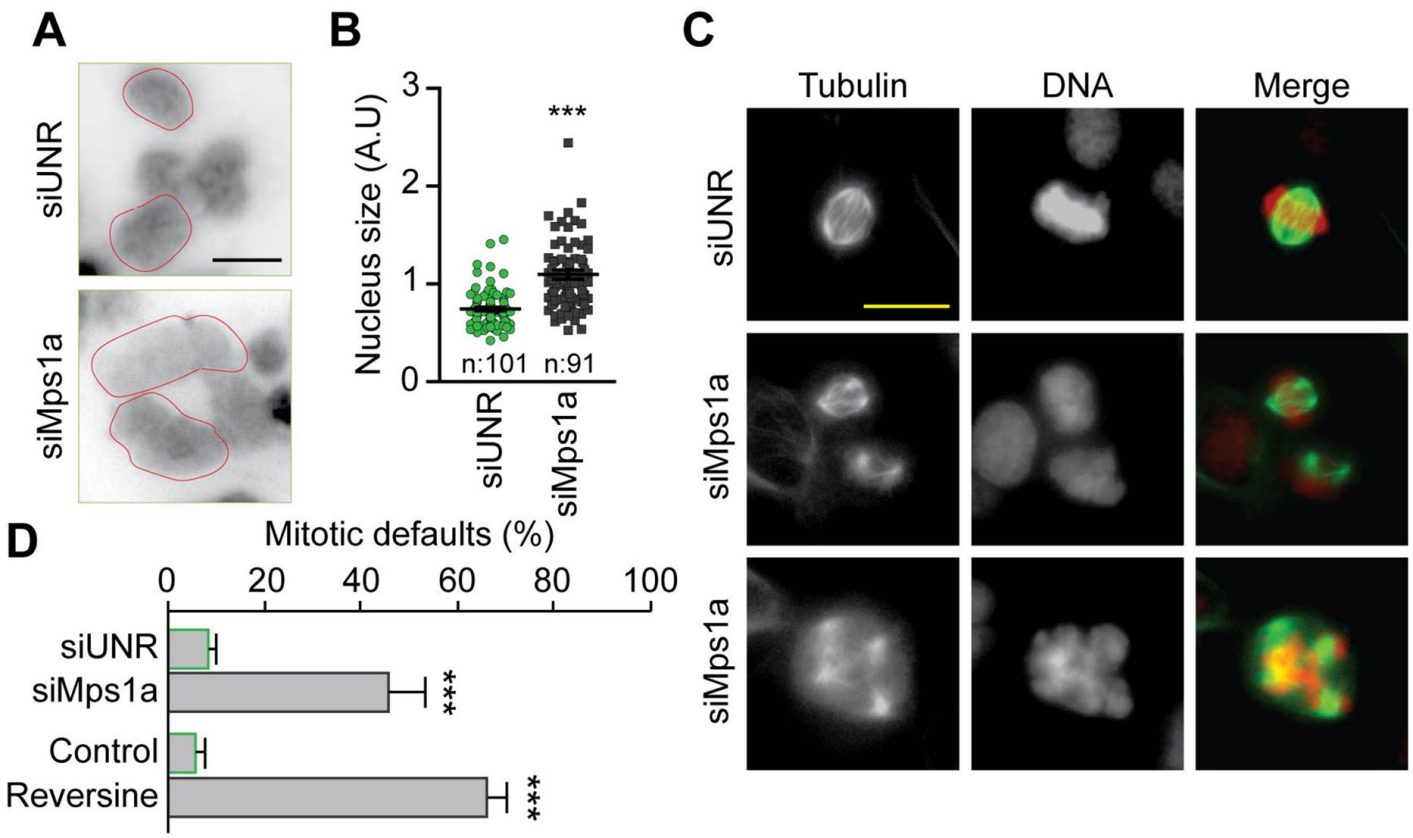
Mps1 inhibition induces mitotic defaults in neuroblastoma cells. (**A**,**B**) SK-N-Be2c neuroblastoma cells were transfected with siUNR or siMps1a for 72 h. Cells were fixed for immunofluorescence and cells nuclei were labeled with DAPI for size estimation. Representative picture of cells nuclei are reported in (**A**) while quantitative data of nucleus size are reported in (**B**). n represents the number of counted nuclei. (**C**,**D**) SK-N-Be2c neuroblastoma cells were treated with DMSO or Reversine, or transfected with siUNR or siMps1a for 72 h. Cells were then fixed for immunofluorescence and labeled with DAPI for DNA detection and anti-α-Tubulin – FITC antibody for the microtubules visualization. For the quantification of mitotic default, mitotic figures were followed. Representative pictures of mitotic default are shown in panel (**C**) while panel (**D**) reports quantitative data for mitotic defaults accumulation. Data are reported in SEM n = 3. ***(p < 0.001) indicates significant difference from control (ANOVA).

When analysing cell cycle profiles by flow-cytometry, we observed that the inhibition of Mps1 provoked a dramatic perturbation of the neuroblastoma cell cycle. SK-N-Be2c neuroblastoma cells undergo polyploidization and subsequent cell death (Fig. 4A). Indeed, cells accumulated high DNA content (> 4*n*) but there was also an increase in subG1 DNA content relative to DNA fragmentation and apoptosis. Similar observations were found in the other neuroblastoma cells tested. To further investigate the cell cycle perturbation after treatment with the Mps1 inhibitors, we evaluated the capacity of the cells to incorporate the thymidine analogue 5-ethynyl-2′deoxyuridine (EdU), an indicator of active DNA synthesis. We found that treated cells showed a decrease in EdU incorporation compared to the control. However, they continue the DNA duplication even when they possessed 4n DNA content (Fig. 4B). Next, we performed an in-depth analysis of the levels of histone H3 phosphorylation, a marker of ongoing mitosis and cell cycling. The cells showed a decline of pH3 after Mps1 inhibitors treatment compared to the control, a sign of accelerated mitosis^37,38^. Nevertheless, we found a two-step of pH3 relative to a 4n and 8n stage (diploid mitosis and tetraploid mitosis) giving a supplementary evidence of cells polyploidization^39^ (Fig. 4C). We then decided to study the effect of Mps1 abrogation on mitosis in SK-N-Be2c neuroblastoma cells. We found that siRNA Mps1 transfected cells also undergo polyploidy, as shown by the increase in the nucleus size compared to cells transfected with siUNR (Fig. 5A,B). Moreover, cells treated with Mps1 inhibitors or transfected with siMps1 exhibited a major disorganisation of mitoses and the accumulation of mitotic defaults. Indeed, cells showed non-aligned metaphase plates, asymmetric chromosome distributions, and increased multipolar spindle organisation, leading to aberrant karyokinesis and/or cytokinesis (Fig. 5C,D). Taken together, these findings suggest that neuroblastoma cells undergo polyploidy upon Mps1 inhibition or silencing. These round of polyploidy engender defective mitosis and non-viable aneuploid daughter cells that die by the activation of the mitochondrial pathway of cell death. This particular death is called mitotic catastrophe^38,40,41^. We found similar results using the neuroblastoma cell lines SK-N-F1, SK-N-AS, IMR32, SK-N-RA and SK-N-DZ treated with Reversine or Mps-BAY2a. Indeed, apoptosis measured with the subG1 fraction (Fig. S3A,B) and the dissipation of the mitochondrial inner transmembrane potential measured with the dye DiOC_6_(3) (see Fig. 2A) was correlated with polyploidy in treated cells (Fig. S3A,B). The observed phenotype was independent of the MYCN, P53 or ALK status of the cells.

### Mps1 inhibition kills patient-derived xenograft cells in vitro and neuroblastoma cells in vivo

**Figure 6:**
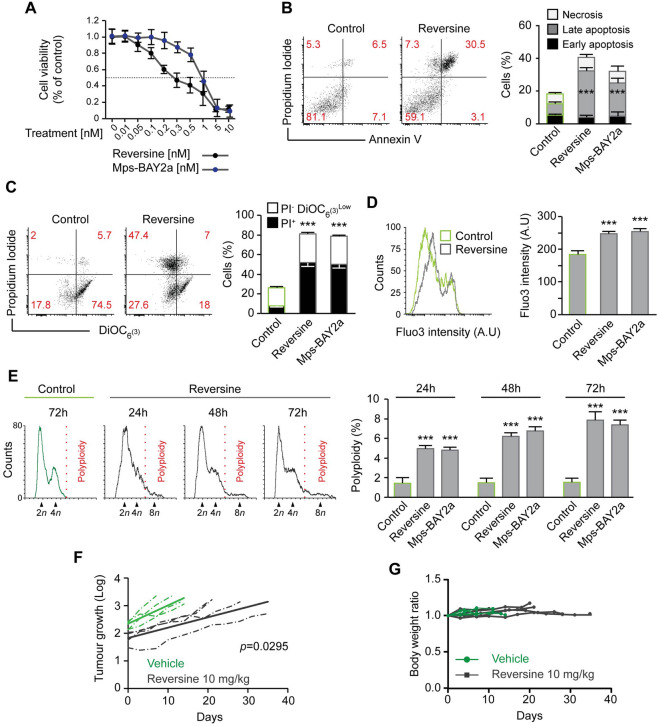
Mps1 inhibition kills PDX cells in vitro and neuroblastoma cells in vivo. (**A**) Cell viability in LU-NB-2 patient-derived xenograft (PDX)-cells treated with increasing concentrations of Reversine or Mps-BAY2a was examined after 72 h using WST-1 assay. (**B**) LU-NB-2 patient-derived xenograft (PDX)-cells were treated for 72 h with DMSO as control or Mps1 inhibitors and then co-stained with the vital dye propidium iodide (PI) and the FITC-conjugated Annexin V for the detection of phosphatidylserine exposure. Representative dot plot (Control vs. Reversine treatment) and quantitative data are reported. Black columns depict the percentage of early apoptotic cells (PI^−^Annexin V^+^), grey columns the percentage of late apoptotic cells (PI^+^Annexin V^+^) and white columns the percentage of necrotic cells (PI^+^Annexin V^−^). Data are indicated as the percentage of cells in each gate. (**C**) LU-NB-2 patient-derived xenograft (PDX)-cells were treated for 72 h with DMSO for the control or Mps1 inhibitors and then co-stained with DiOC_6_(3), and propidium iodide (PI) for the assessment of apoptosis-associated parameters. Representative dot plot (Control vs. Reversine treatment) and quantitative data are reported. Black columns depict the percentage of dead (PI^+^) cells and white columns the percentage of dying (PI^−^ DiOC6(3)^low^) cells. Data are represented as the percentage of cells in each gate. (**D**) LU-NB-2 patient-derived xenograft (PDX)-cells were stained with the calcium dye Fluo-3 for quantification of the cytosolic Ca^2+^ concentration by flow cytometry. Representative histogram of Reversine vs. DMSO treated cells and quantitative data of the dye intensity are presented. Control cells treated with DMSO are depicted in green and Mps1 inhibitors treated groups in grey. (**E**) LU-NB-2 patient-derived xenograft (PDX)-cells were treated with DMSO or Mps1 inhibitors for 24 h, 48 h and 72 h. Cells were labelled for cell cycle analysis. Representative histograms (Control vs. Reversine) and quantitative data of polyploid fraction are reported. (**F**,**G**) In vivo growth arrest of SK-N-Be2c neuroblastoma cells by Reversine. Cells were injected subcutaneously into athymic mice (4 mice for Reversine-treated groups, 4 mice for vehicle-treated controls), and when a tumour was observed intraperitoneal injections was performed, as described in Materials and Methods Section. Tumour size (**F**) and animal weight (**G**) was then routinely monitored by means of a common calliper. The slope of all vehicle vs. Reversine-treated tumours curve was calculated to access the statistic relevance of the treatment. Data are reported in SEM n = 3. ***(p < 0.001) indicates significant difference from control (ANOVA). In vivo statistical significance was determined by log transforming tumour volume at each time point followed by analysis of covariance between groups.

To further confirm the therapeutic advantage of Mps1 inhibition in neuroblastoma, we decided to use the neuroblastoma patient-derived xenograft (PDX) model. Indeed, PDX models conserve the biological features of the original tissue and are recognised as accurate and clinically relevant models^42^. For this purpose, we used the LU-NB-2 patient-derived xenograft cells^43^, which were extracted from a metastatic tumour, resistant to chemotherapy treatment. These cells have MYCN amplification but no evidence of p53 or ALK mutation^43,44^. We initially performed a dose–response viability assay using WST-1 test with Reversine and Mps-BAY2a. It was found that the LU-NB-2 cells were sensitive to the Mps1 inhibitors in a dose-dependent manner (Fig. 6A). To characterize the cell death pathway engaged in the LU-NB-2 cells, we treated these cells with Reversine and Mps-BAY2a in comparison to control (DMSO) for 72 h. Cells were further co-stained with the vital dye PI and the apoptotic marker Annexin-V. LU-NB-2 cells showed to undergo apoptosis after Mps1 inhibition (Fig. 6B). To further confirm this finding, we performed another apoptotic assay, the co-staining with the mitochondrial inner transmembrane potential (Δψm) sensitive dye DiOC_6_(3) and the vital dye propidium iodide (PI) in addition to the measurement of the intracellular calcium (Ca^2+^) concentration. Mps1 inhibition promoted dissipation of the mitochondrial membrane potential and an increase of intracellular calcium concentration (Fig. 6C,D). Moreover, co-treatment with the pan-caspase inhibitor Z-VAD-fmk reduced the toxicity of Mps1 inhibitors (Figure S4A). This finding confirms the mitochondrial pathway of apoptosis as main cell death subroutine engaged after Mps1 inhibition in patient-derived xenograft LU-NB-2 cells. Similar to SK-N-Be2c, we also found that Mps1 inhibition does not induce any necroptosis or autophagy in LU-NB-2 cells (Figure S4A,B). Cell cycle profile by flow-cytometry analysis demonstrated that LU-NB-2 cells undergo polyploidy in a time-dependent manner after Mps1 inhibition (Fig. 6E). Altogether, our data demonstrate that in LU-NB-2 PDX cells, Mps1 inhibition induces mitotic catastrophe. We then decided to evaluate the effect of Mps1 inhibition in vivo on SK-N-Be2c neuroblastoma. The intraperitoneal administration of Reversine as Mps1 inhibitor affected significantly the growth of the xenografted tumours in athymic nude mice compared to the tumours treated with vehicle (Fig. 6F). Moreover, the doubling time of the tumour size was 4.15 days for the vehicle treated group and 14.29 days for the Reversine treated one. This gives a supplementary evidence for tumour growth delay in a xenograft mouse model following treatment with Reversine. In addition, there were no differences in the average body weight when comparing the vehicle and Reversine-treated mice showing the potential in vivo tolerability of Reversine (Fig. 6G). Altogether, these data underscore the advantage of Mps1 inhibition as a preclinical model for neuroblastoma.

## Discussion

Treating patients with high-risk neuroblastoma remains challenging, as shown by the limit of actual treatments and the risk of toxicity following intensive therapies^11^. The use of anti-mitotic drugs/agents is an attractive approach in cancer therapies, in particular when targeting cell cycle players^45–47^. High-level mitotic kinase expression has previously been reported in a variety of paediatric cancers and in particular high-risk neuroblastomas^48–50^. Here, we propose mitotic kinase Mps1 as a target for neuroblastoma cells regardless of MYCN copy number status.

We evaluated how Mps1 gene expression (TTK) is regulated in cohorts of neuroblastoma patients and to what degree this could correlate with patient prognosis. We found that among all data sets we analysed (4 showed in this study), Mps1 overexpression is highly correlated with advanced stages of the tumour and MYCN amplification, which are prediction markers for poor outcomes. Moreover, Mps1 overexpression was associated with poor overall and relapse-free survival as showed by the Kaplan–Meier curves. These data strongly support Mps1 overexpression as a novel prognostic marker for human neuroblastoma. Mps1 kinase is an emergent target in cancer therapy, and several studies have focused on its role as the “Achilles heel” of tumours especially in colon carcinoma, breast cancer, sarcoma, ovarian cancer and glioblastoma^21,24,25,38,51,52^.

Here, we developed a strategy to target neuroblastoma cells by the inhibition of Mps1 kinase. By employing different neuroblastoma cell lines and a patient-derived xenograft (PDX) model, we provided strong evidence that the depletion or inhibition of Mps1 effectively kill these cells. These results were confirmed in vivo using xenograft tumours in athymic nude mice.

The observed cell death is executed by the induction of mitotic catastrophe followed by aberrant cell divisions and a consequent polyploidy and aneuploidy cascade, and the activation of a the mitochondrial and caspase-dependent pathway of apoptosis. Upon exposure to Mps1 inhibitors, Reversine or Mps-BAY2a, or the silencing of this kinase using specific siRNAs, neuroblastoma cells displayed severely abnormal anaphases, and as they progressed in mitosis, they underwent the polyploid-aneuploid cascade. Illicit multipolar and/or bipolar divisions follow this catastrophic fate. The generated polyploid cells or aneuploid daughter cells undergo cell death due to the accumulation of chromosomal instability. This particular cell death, known as mitotic catastrophe^40,53^, is an oncosuppressive mechanism for the control of mitosis-incompetent cells. As shown in our models, the mitochondrial pathway of apoptosis drives this subroutine of apoptosis. Indeed, neuroblastoma and PDX cells displayed mitochondrial potential dissipation, the accumulation of cytosolic calcium, activation of the caspase-3, cleavage of PARP, and DNA degradation. These results are in line with previous studies with Mps1 inhibitors or Mps1 silencing confirming the key role of this kinase in the spindle assembly checkpoint function and its vital function, both in vitro and in vivo^20,21,24,25,35,38,39,51,52,54–58^.

In conclusion, we demonstrated here that Mps1 depletion or inhibition kills high-risk neuroblastoma cells in vitro and in vivo. Our study proposes new windows in the context of cancer therapy given that these paediatric tumours display high resistance to conventional anticancer regimens such as DNA damaging agents^59,60^.

## Material and methods

### Animals

All animals were maintained under specific pathogen-free conditions at the Medicon Village animal facility at Lund University. All animal experiments were performed according to the national and international guidelines of the European Union. Furthermore, all animal experiments were approved by the Swedish regional (Malmö-Lund) ethical committee with ethical number M129-15.

### Cell lines and culture conditions

Media and supplements for cell culture were purchased from HyClone (Thermo Fisher). Cell lines were routinely maintained at 37 °C under 5% CO_2_. Media were supplemented with 10% FBS (Sigma, Germany) and 0.1% penicillin/streptomycin (GIBCO, USA). The human neuroblastoma cell lines SK-N-Be2c, SK-N-F1, SK-N-SH and IMR32 were cultured in MEM. SK-N-DZ cells were cultured in DMEM. SK-N-AS cells were cultured in DMEM supplemented with 0.1 mM Non-Essential Amino Acids (NEAA). SK-N-RA cells were cultured in RPMI.

LU-NB-2 patient-derived xenograft (PDX)-cells were established and characterized as previously described^43^. Briefly, PDX cells were derived from a NB brain metastasis following treatment relapse. PDX cells were cultured as free-floating 3D-spheres in serum-free medium consisting of Dulbecco’s Modified Eagle’s Medium (DMEM) and GlutaMAXTM F-12 (3:1 ratio) supplemented with 1% penicillin/streptomycin, 2% B27 w/o vitamin A, 40 ng/µl basic Fibroblast Growth Factor and 20 ng/µl Epidermal Growth Factor.

### Chemicals

Reversine (BioNordika, Stockholm, Sweden), Mps-BAY2a (Tocris Bioscience, Bristol, UK), were stocked as 10 mM solution in DMSO. The pan-caspase inhibitor z-VAD-fmk (Sigma-Aldrich, Germany) was stocked as a 50 mM. The necroptosis inhibitor Necrostatine-1 (Sigma-Aldrich, Germany) was stocked as a 100 mM. The appropriate amount of DMSO or ethanol was always employed for negative control conditions.

### Quantitative PCR

The cells were rinsed in cold PBS and total RNA was extracted using the RNeasy Mini kit (Qiagen, #74106) and performed according to manufacturer’s instruction. The purity of RNA was analyzed and quantified by a NanoDrop 2000 spectrophotometer (Thermo Fisher) and used for cDNA synthesis according to the manufacturer’s instruction (High Capacity cDNA Reverse transcription kit #4368814, Applied Biosystems). PCR runs were performed in the QuantStudioTM 7 Flex System using SYBR^®^Green Reagent (Applied Biosystems), with the following program; 2 min 50 °C, 10 min in 95 °C followed by 40 three-step cycles consisting of 95 °C for 20 s and 60 °C for 30 s and 72 °C for 1 min. We used the following primers to access Mps1 mRNA.

- Forward primer: TCAAGGAACCTCTGGTGTCA.
- Reverse primer: GGTTACTCTCTGGAACCTCTGGT.

We used the following primers to access actin mRNA.

- Forward primer: AGAGCTACGAGCTGCCTGAC.
- Reverse primer: AGTACTTGCGCTCAGGAGGA.

### RNA interference

Cells were seeded at low density in 6-well plates and after 24 h transfected with a non-targeting siRNA (siUNR) with a sequence unrelated to the human genome (UNR, sense 5′-GCCGGUAUGCCGGUUAAGUdTdT-3′) or with 3 siRNAs directed against Mps1 mRNAs (all purchased from Eurogentec, Liege) by means of oligofectamine RNAiMAX transfection reagent (Thermo Fisher Scientific-Invitrogen), according to the manufacturer’s instructions. After 48 h, transfection efficiency was determined by immunoblotting.

The following Mps1 siRNAs were used:

- siMps1a sense 5′-CCCAGAGGACUGGUUGAGUdTdT-3′.
- siMps1b sense 5′-GCAACCACUUAUGGUACUGdTdT-3′.
- siMps1c sense 5′-GCACGUGACUACUUUCAAAdTdT-3′.

### Cytofluorometric studies

Cytofluorometric acquisitions were performed by means of a FACSVerse cytofluorometer (BD Biosciences). Data were statistically evaluated using the FCS Express 6 Flow (De Novo Software, CA, USA) software.

#### Measurement of cell shrinkage

For the quantification of cell shrinkage, cells were harvested and collected with the culture medium before FACS assessment without any staining. We measured the cell size using the forward scatter FSC parameter. Apoptotic and shrinked cells are smaller than live cells.

#### Measurement of cell scrambling and phosphatidylserine exposure

For the simultaneous quantification of plasma membrane integrity and Phosphatidylserine exposure, cells were harvested and collected with the culture medium and stained with Annexin-V-FITC (1:200 dilution; ImmunoTools, Friesoythe, Germany) and 1 μg/ml propidium iodide (PI, which only incorporates into dead cells, from Sigma-Aldrich) for 30 min at 37 °C before FACS assessment.

#### Measurement of mitochondrial membrane potential

For the simultaneous quantification of plasma membrane integrity and mitochondrial transmembrane potential (Δψm), cells were harvested and collected with the culture medium and stained with 1 μg/ml propidium iodide and 40 nM 3,3′-dihexyloxacarbocyanine iodide (DiOC6(3), a Δψm-sensitive dye) (Molecular Probes-Invitrogen, Eugene, OR, USA) for 30 min at 37 °C before FACS assessment.

#### Measurement of mitochondria accumulation

For staining mitochondria, cells were harvested and collected with the culture medium and labelled during 45 min at 37 °C with 100 nM of the MitoTracker Red MTR (Thermo Fisher) before FACS assessment. The signal shift is measured comparatively to non-treated cells.

#### Measurement of intracellular calcium concentration

For the evaluation of the cytosolic Ca^2+^, cells were collected and suspended in growth medium loaded with 5 μM of the calcium tracker Fluo-3/AM (Biotium, Hayward, CA, USA). The cells were incubated at 37 °C for 30 min before Ca^2+^-dependent fluorescence intensity measurement. The Fluo-3 AM is measured with an excitation wavelength of 488 nm (blue laser) and an emission wavelength of 530 nm. The signal shift and the Geo Mean are measured comparatively to non-treated cells.

#### Measurement of caspase-3 activation

To measure the level of cleaved caspase-3, cells were fixed with 75% (v/v) ethanol in PBS, permeabilized with 0.25% (v/v) Tween 20 in PBS and stained overnight with a rabbit antiserum specific for active caspase-3 coupled with the fluorochrome FITC (FITC Rabbit Anti-Active caspase-3, Clone C92-605 #559341, BD Biosciences).

#### Measurement of cleaved PARP

To measure the level of cleaved PARP, cells were fixed with 75% (v/v) ethanol in PBS, permeabilized with 0.25% (v/v) Tween 20 in PBS and stained overnight with a rabbit antiserum specific for cleaved PARP (Rabbit anti PARP cleaved (Y34), #ab32561, Abcam).

#### Cell cycle analysis

For the quantification of cell cycle profiling (DNA content), live cells were harvested and collected with the culture medium and re-suspended in 0.3 ml pre-warmed growth medium supplemented with 2 mM Hoechst 33342 (Sigma-Aldrich, Germany) for 30 min at 37 °C in a 5% CO_2_ incubator. Cells suspensions were analysed on the cytometer, with ultraviolet excitation and emission collected at > 450 nm. To quantify the apoptotic and DNA fragmented fraction we measure the subG1 population of the cell cycle.

#### Measurement of autophagy

To measure autophagy, we used Acridine Orange (Thermo Fisher) staining and the acidic vesicular organelles (AVOs) as hallmark of autophagy. Cells were trypsinized, collected and stained with 1 μg/ml Acridine Orange (2.7 μM) in complete culture medium for 15 min at room temperature before FACS acquisition in the presence of the staining solution. The red-to-green fluorescence ratio is considered to quantify the development of AVOs^36^.

#### Measurement of cells cycle markers pH3

To measure histone H3 phosphorylation on serine 10, cells were fixed with 75% (v/v) ethanol in PBS, permeabilized with 0.25% (v/v) Tween 20 in PBS and stained overnight with a rabbit antiserum specific for phosphorylated histone H3 (rabbit polyclonal IgG1 #06–570, Millipore-Chemicon International).

#### Measurement of EdU incorporation

To measure EdU incorporation, cells were incubated with 10 μM EdU for 30 min at 37 °C, fixed, permeabilized and stained with the Fluorescent dye azide (Click-iTTM reaction cocktail, #C10425, Invitrogen) and DAPI according to the manufacturer’s instructions.

### Immunofluorescence and confocal microscopy

Cells were cultured on coverslips in 6-well plates for 24 h and then fixed in 100% methanol for 10 min at − 20 °C. Next, the cells were incubated in 10% FBS-PBS for 1 h at room temperature with anti-α-Tubulin-FITC antibody, (Clone DM1A, # F2168-0.2ML. Sigma-Aldrich, Germany) and DAPI for DNA staining. Then the coverslips were washed and mounted in Fluorescence Mounting medium (Dako, Santa Clara, CA, USA). The images were obtained using a Zeiss LSM710 confocal microscope.

### WST-1 cell viability assay

LU-NB-2 patient-derived xenograft (PDX) cells were seeded in 96-well plates (3,000 cells/well/100 μl) and treated with increasing concentrations of Reversine or Mps-BAY2a (0.01, 0.05, 0.1, 0.2, 0.3, 0.5, 1, 5 and 10 μM). After 72 h, WST-1 reagent (Roche Applied Science, Mannheim, Germany) was subsequently added (10 μl) to each well and cells were incubated for 4 h at 37 °C before measuring the absorbance. Each well was measured at a wavelength of 450 nm and reference wavelength of 690 nm, using a scanning multi-well spectrophotometer (Synergy 2). Each experiment was performed in triplicate and repeated three times.

### In vivo xenograft models

6-week-old mice NMRI-nu/NMRI-Foxn1nu/nu (body weight 27 g, provided by the Janvier Labs) were used throughout this study in strict compliance with the Malmö and Lund Animal Ethics Committee (ethical number M129-15). The animals were maintained under specific pathogen-free (SPF) conditions and were provided with food and water ad libitum. Light cycle was artificially controlled to provide 14 h of light. After an acclimation period, mice were subcutaneously xenografted with 2 × 10^6^ SK-N-Be2c cells in 0.1 ml PBS. When a tumour was observed, mice were injected i.p. with 10 mg/kg Reversine (or an equivalent volume of vehicle) twice per week. Due to its limited solubility in water, Reversine was injected in 2% DMSO, 2% polyethylene glycol 600 (PEG600) and 2% Tween 80 in PBS.

### Statistical analyses

Statistical analyses were performed using the GraphPad Software (San Diego, CA, USA). P-values < 0.05 were deemed statistically significant. Statistical comparisons were assessed by ANOVA (Kruskal–Wallis test). In vivo statistical significance was determined by log transformation of the tumour volumes at each time point followed by analysis of covariance between groups.

## Supplementary information

Supplementary Information.
