# Supplementary material for: Inhibition of mitotic kinase Mps1 promotes cell death in neuroblastoma
Source: Sci Rep. 2020 Jul 20;10:11997. doi: 10.1038/s41598-020-68829-y (PMC7371706; doi:10.1038/s41598-020-68829-y)
Supplement: Supplementary file 1 — Supplementary Information. [file 41598_2020_68829_MOESM1_ESM.pdf]

Supplementary Information

Inhibition of mitotic kinase Mps1 promotes cell death in Neuroblastoma

Sonia Simon Serrano, Wondossen Sime, Yasmin Abassi, Renée Daams,
Ramin Massoumi* and Mohamed Jemaà*

Department of Laboratory Medicine, Translational Cancer Research, Faculty of Medicine, Lund University, Lund 22381, Sweden.

* R.M. and M.J. share senior co-authorship

Correspondence to:

Dr. Mohamed Jemaà (jemaamohamed@gmail.com / mohamed.jemaa@med.lu.se) and Dr. Ramin Massoumi (ramin.massoumi@med.lu.se) Department of Laboratory Medicine, Translational Cancer Research, Lund University, Lund 22381, Sweden Tel. Tel. +46 46 222 64 30.

Figure S1. Mps1 expression predicts clinical outcome in neuroblastoma patients.

A. Box plots of Mps1 expression relative to tumor stage, MYCN status followed by Kaplan–Meier curves reporting patients' event-free survival and overall survival probability with respect to Mp1 expression extracted from data set GSE45547.

B. Box plots of Mps1 expression relative to tumor stage, MYCN status followed by Kaplan–Meier curves reporting patients' event-free survival and overall survival probability with respect to Mp1 expression extracted from data set GSE16476.

C. Box plots of Mps1 expression relative to tumor stage, MYCN status followed by Kaplan–Meier curves reporting patients' event-free survival and overall survival probability with respect to Mp1 expression extracted from data set E-TABM-38.

Figure S2. Mps1 inhibition does not induce necroptosis or autophagy in neuroblastoma cells.

A. SK-N-Be2c neuroblastoma cells were treated for 72h with DMSO as control (framed in green), 0.3 μM Reversine (framed in orange) or 1 μM Mps-BAY2a (framed in blue) followed by flow cytometry acquisition. The events characterized by normal forward scatter (FSC) and side scatter (SSC) in the control cells were considered as a reference. Shrinkage of the cells, characterized by a decrease in cell size (FSS), is considered a hallmark of apoptosis. Representative histograms of forward scatter with the gate FSC< 80 and quantitative data are reported.

B. Effects of the necroptosis inhibitor necrostatin-1 on Mps1 inhibitors induced cell death. SK-N-Be2c neuroblastoma cells were treated for 72h with Mps1 inhibitors alone or in combination with 50 μM or 100 μM Necrostatin-1 followed by $DiOC_6(3)$/PI co-staining. Quantitative data are represented. White and black columns depict the percentage of dying ($PI^{-}DiOC6(3)^{low}$) and dead (PI^{+}) cells, respectively.

C. Cells were treated for 72 h with DMSO as control, 0.3 μM Reversine or 1 μM Mps-BAY2a and then stained with Acridine Orange to quantify autophagy by flow cytometry. Representative dot plot (Reversine treatment) of Red and green fluorescence of Acridine-Orange-stained cells are reported. Values represent the percentage above the Red/Green ratio that refer to autophagic population. Control cells treated with DMSO are depicted in green while Mps1 inhibitors treated ones are depicted in grey.

Data are reported in SEM n = 3.

Data are reported in SEM n = 3. ***($p<0.001$) indicates significant difference from the DMSO control treatment (ANOVA).

Figure S3. Polyploidy is correlated with death induced by Mps1 inhibition in neuroblastoma cells.

A-B. Neuroblastoma cells SK-N-F1, SK-N-AS, IMR32, SK-N-RA and SK-N-DZ cells were cultured in the presence of DMSO, Reversine or Mps-BAY2a for up to 72h. Cells were fixed with cold 75% ethanol and labeled with propidium iodide (PI) as DNA dye, for the quantification of the cell cycle. Representative histograms are reported in panel (**A**) while quantitative data of polyploid and subG1 fraction are reported in panel (**B**). Data are reported in SEM n = 3. **($p<0.01$) and ***($p<0.001$) indicate significant differences from the DMSO control treatment (ANOVA).

Figure S4. Mps1 inhibition does not induce necroptosis or autophagy in LU-NB-2 patient-derived xenograft (PDX)-cells.

A. Effects of the poly-caspase inhibitor Z-VAD-fmk and necroptosis inhibitor necrostatin-1 on Mps1 inhibitors induced cell death.

LU-NB-2 patient-derived xenograft (PDX)-cells were treated for 72h with Mps1 inhibitors alone or in combination with 50 µM Z-VAD-fmk or 100 µM Necrostatin-1 followed by $DiOC_6(3)$/PI co-staining. Quantitative data are represented. White and black columns depict the percentage of dying ($PI^- DiOC_6(3)^{low}$) and dead (PI^+) cells, respectively.

B. Cells were treated for 72 h with DMSO as control, 0.3 µM Reversine or 1 µM Mps-BAY2a and then stained with Acridine Orange to quantify autophagy by flow cytometry. Representative dot plot (Reversine treatment) of Red and green fluorescence of Acridine-Orange-stained cells are reported. Values represent the percentage above the Red/Green ratio that refer to autophagic population. Control cells treated with DMSO are depicted in green while Mps1 inhibitors treated groups are depicted in grey.

Data are reported in SEM n = 3.

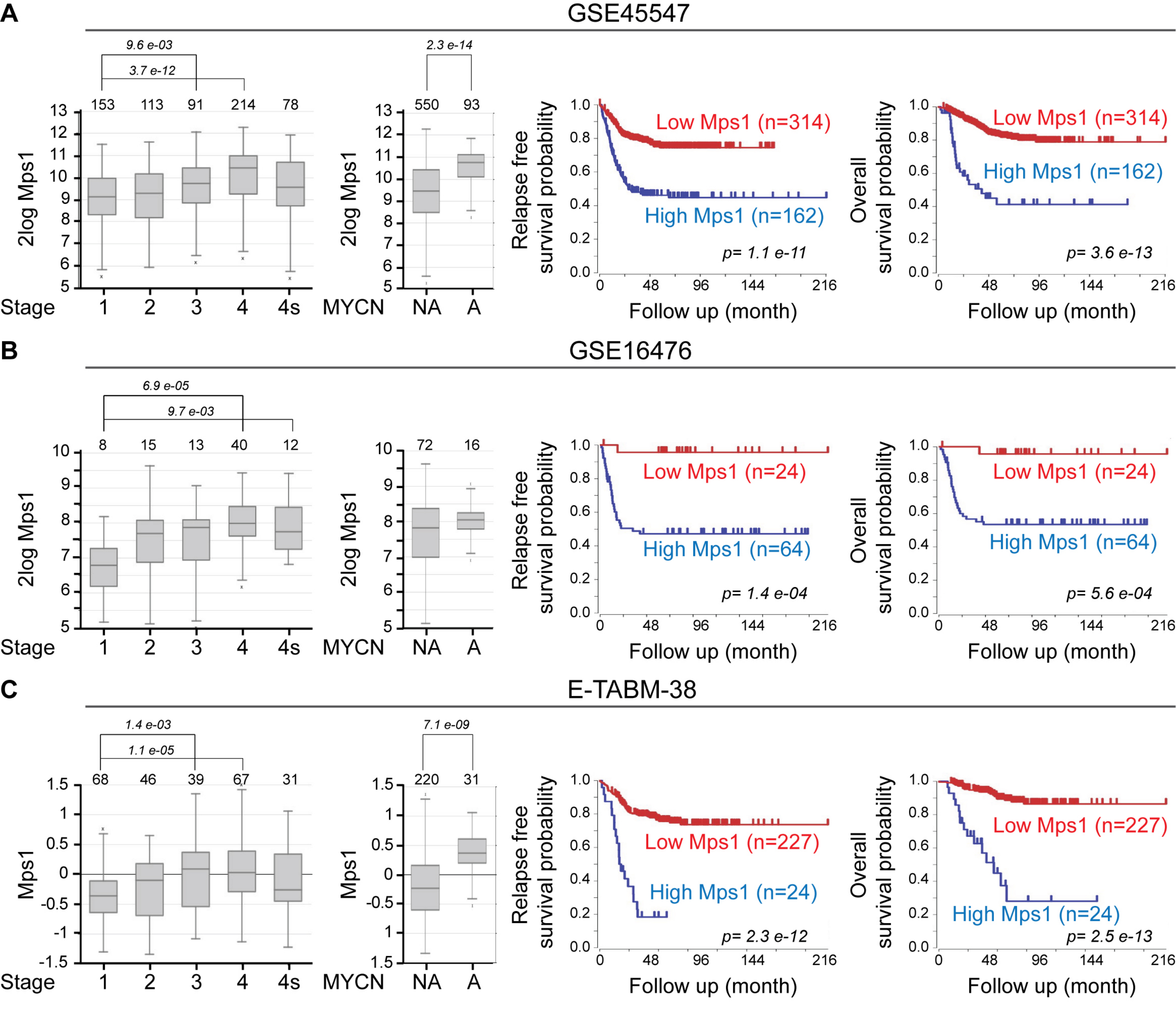
A
GSE45547
9.6 e-03
3.7 e-12
153 113 91 214 78
2log Mps1
Stage 1 2 3 4 4s
2.3 e-14
550 93
MYCN NA A
Relapse free survival probability
Low Mps1 (n=314)
High Mps1 (n=162)
p= 1.1 e-11
Follow up (month)
Overall survival probability
Low Mps1 (n=314)
High Mps1 (n=162)
p= 3.6 e-13
B
GSE16476
6.9 e-05
9.7 e-03
8 15 13 40 12
72 16
Low Mps1 (n=24)
High Mps1 (n=64)
p= 1.4 e-04
p= 5.6 e-04
C
E-TABM-38
1.4 e-03
1.1 e-05
68 46 39 67 31
Mps1
7.1 e-09
220 31
Low Mps1 (n=227)
High Mps1 (n=24)
p= 2.3 e-12
p= 2.5 e-13

FIGURE S1

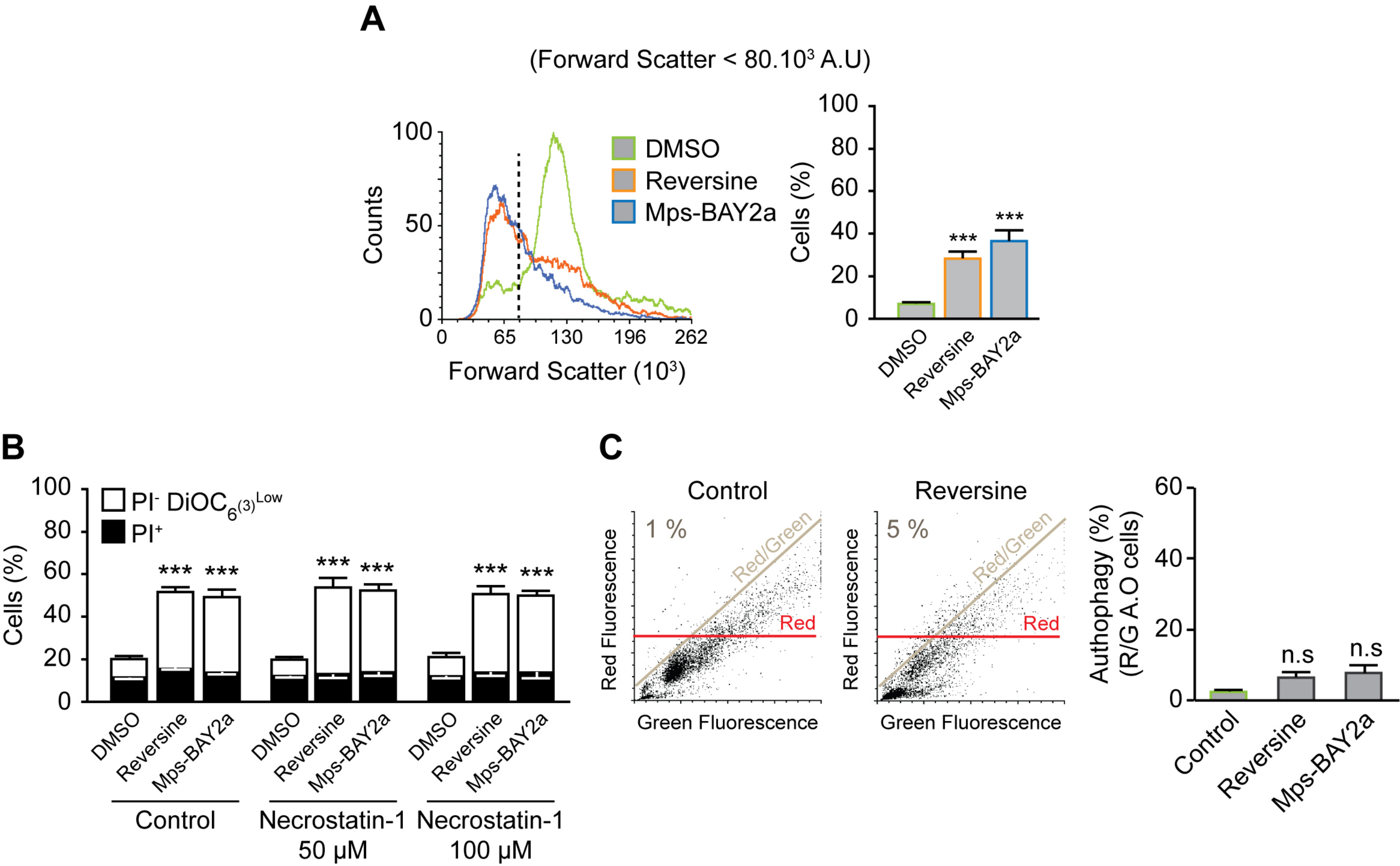
A
(Forward Scatter < 80.10³ A.U)
100
50
0
Counts
0
65
130
196
262
Forward Scatter (10³)
DMSO
Reversine
Mps-BAY2a
100
80
60
40
20
0
Cells (%)
***
***
DMSO
Reversine
Mps-BAY2a
B
100
80
60
40
20
0
Cells (%)
PI⁻ DiOC6(3) Low
PI⁺
***
***
***
***
***
***
DMSO
Reversine
Mps-BAY2a
DMSO
Reversine
Mps-BAY2a
DMSO
Reversine
Mps-BAY2a
Control
Necrostatin-1 50 µM
Necrostatin-1 100 µM
C
Control
Reversine
1 %
5 %
Red/Green
Red/Green
Red
Red
Red Fluorescence
Red Fluorescence
Green Fluorescence
Green Fluorescence
60
40
20
0
Authophagy (%)
(R/G A.O cells)
n.s
n.s
Control
Reversine
Mps-BAY2a

FIGURE S2

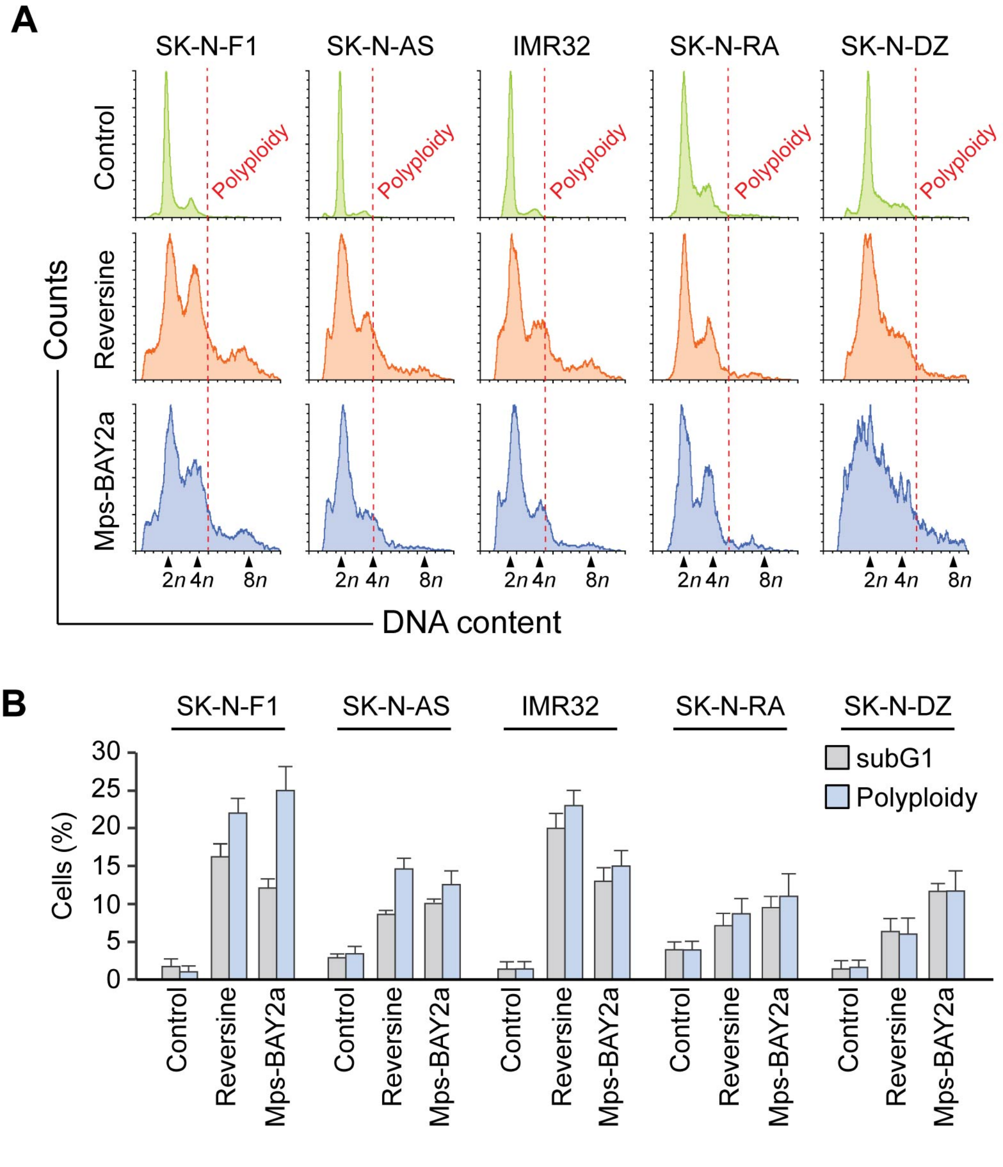
A
SK-N-F1
SK-N-AS
IMR32
SK-N-RA
SK-N-DZ
Control
Reversine
Mps-BAY2a
Polyploidy
Counts
2n 4n 8n
DNA content
B
subG1
Polyploidy
Cells (%)
30
25
20
15
10
5
0
Control
Reversine
Mps-BAY2a

FIGURE S3

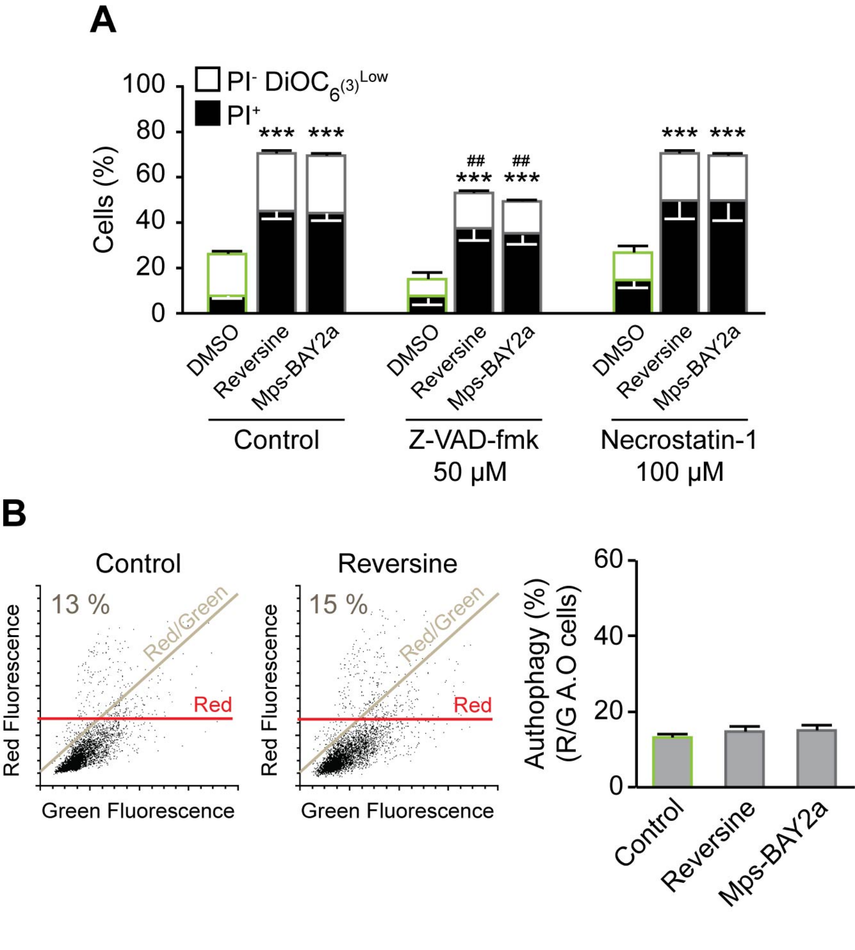
A
PI⁻ DiOC6(3) Low
PI+
Cells (%)
100
80
60
40
20
0
***
***
##
***
##
***
***
***
DMSO
Reversine
Mps-BAY2a
DMSO
Reversine
Mps-BAY2a
DMSO
Reversine
Mps-BAY2a
Control
Z-VAD-fmk
50 µM
Necrostatin-1
100 µM
B
Control
Reversine
13 %
15 %
Red/Green
Red
Red Fluorescence
Green Fluorescence
Authophagy (%)
(R/G A.O cells)
60
40
20
0
Control
Reversine
Mps-BAY2a

FIGURE S4
